# Exploring factors associated with hepatitis B screening in a multilingual and diverse population

**DOI:** 10.1186/s12913-022-07813-w

**Published:** 2022-04-11

**Authors:** Janet N. Chu, Tung T. Nguyen, Natalie A. Rivadeneira, Robert A. Hiatt, Urmimala Sarkar

**Affiliations:** 1 Division of General Internal Medicine, Department of Medicine, University of California San Francisco, 1545 Divisadero Street, Suite 322, San Francisco, CA 94115 USA; 2 Center for Vulnerable Populations, Zuckerberg San Francisco General Hospital, University of California San Francisco, San Francisco, California USA; 3 Department of Epidemiology and Biostatistics, University of California San Francisco, San Francisco, California USA; 4 Helen Diller Family Comprehensive Cancer Center, University of California San Francisco, San Francisco, California USA

**Keywords:** Hepatitis B, Screening, Language preference, Race/ethnicity, Preventive medicine

## Abstract

**Background:**

Racial/ethnic minorities bear a disproportionate burden of hepatitis B virus (HBV) infection and disease. Disparities in HBV screening contribute to worse outcomes for communities of color. We examined the impact of race/ethnicity, language preference, and having a usual place of care on HBV screening in a multilingual, urban cohort.

**Methods:**

We used questions from the Health Information National Trends Survey and added validated questions about healthcare access and health literacy. We administered this survey in English, Spanish, and Chinese to a selected convenience sample of San Francisco city/county residents in 2017, with pre-specified targets for populations with known cancer disparities: 25% Spanish-speaking, 25% Chinese-speaking, and 25% Black Americans. Using weighted multivariable logistic regression analyses, we assessed how race/ethnicity, language preference, and having a usual place of care impacts self-report of HBV screening.

**Results:**

Overall, 1027 participants completed the survey (50% of surveys administered in English, 25% in Spanish, and 25% in Chinese). Only 50% of participants reported HBV screening. In multivariable analysis, Black (OR = 0.20, 95% CI 0.08–0.49), Latinx (OR = 0.33, 95% CI 0.13–0.85), Asian (OR = 0.31, 95% CI 0.10, 0.94), and ‘Other’ race/ethnicity (OR = 0.17, 95% CI 0.05–0.53) respondents had lower odds of HBV screening compared to non-Hispanic White respondents. Participants who had insurance had increased odds of HBV screening (OR = 2.70, 95% CI 1.48–4.93).

**Conclusions:**

HBV screening disparities persist for Black Americans, Asian Americans, Latinx, and the uninsured. Future studies should explore reasons why current strategies have not been implemented or are not successful, particularly in addressing racial/ethnic and insurance disparities.

**Supplementary Information:**

The online version contains supplementary material available at 10.1186/s12913-022-07813-w.

## Background

Prior studies have shown disparities in chronic hepatitis B (HBV) infection and related complications, with higher prevalence among African American, Asian American, and Native Hawaiian Pacific Islander communities compared to White populations [1], and foreign-born individuals compared to those born in the United States (U.S.) [2]. To address disparities in HBV care, individuals infected with HBV need to be accurately identified and appropriately linked to care. HBV screening can prevent morbidity and mortality related to chronic liver disease and liver cancer. HBV screening leads to individual awareness about hepatitis status, prevents transmission of HBV by those unaware of their infectivity, promotes vaccination for the uninfected to prevent HBV infection, and enables those infected to seek timely care, including treatment, if appropriate [3]. However, in a national survey, 66% of people with HBV were unaware of being infected [4]. Documented barriers to screening include low rates of public awareness, individuals not perceiving themselves to be at high risk, linguistic and cultural barriers, low socioeconomic status, and lack of health insurance [5–7]. Gaps in physician knowledge regarding HBV screening and management recommendations as well as limited health insurance coverage for HBV screening tests also contribute to low screening rates in the U.S. [8]

We aim to explore the association of race/ethnicity, language preference, and access to care on HBV screening in a sample of multilingual, diverse, low-income participants. Understanding factors associated with HBV screening can lead to targeted interventions to reduce disparities in HBV-related outcomes (e.g., cirrhosis, hepatocellular cancer) in these vulnerable populations.

## Methods

### Survey development and administration

Drawing from questions from the Health Information National Trends Survey (HINTS) [9], a national population-based survey administered by the National Cancer Institute (NCI), we added validated questions about health care access [10] and health literacy [11, 12] to create a local survey (see Additional file 1). We described the development and translation of the full survey and its administration in greater detail elsewhere [13]. Briefly, we administered the survey in the City and County of San Francisco in 2017. We used a community-based sampling approach to optimize survey recruitment from populations likely to have liver disease and who bear the burden of cancer disparities; specifically, we had prespecified targets for race/ethnicity (25% Black American) and language (50% English, 25% Spanish, 25% Chinese) [13]. We worked with San Francisco Cancer Initiative (SF CAN), a local collaborative ‘collective impact’ effort to reduce cancer burden in San Francisco [14], as well as several local community-based organizations to identify community events and popular community establishments at which to recruit participants. Participants included adults who were 18 to 75 years old, lived in San Francisco, and able to complete the survey in English, Spanish, or Chinese. Trained bilingual staff administered the survey in person in the participants’ preferred language (English, Spanish, or Chinese). Participants provided informed written consent. Consent was reviewed at the start of each participant survey. The University of California San Francisco Institutional Review Board approved this study (16–20,707). All methods were performed in accordance with relevant ethical guidelines and regulations of the Declaration of Helsinki.

### Variables

The outcome of interest was self-reported prior HBV screening, defined as participants answering ‘Yes’ to the validated question: ‘Have you ever had a blood test to check for hepatitis B?’ We used the Health Behavior Framework (see Additional file 2) to identify a list of potential factors that could explain variations in HBV screening rates. The predictors of interest were race/ethnicity, language preference, and a usual place of care. Other variables included age, gender, education, English proficiency, health literacy, and insurance status.

Age (i.e. 18–34 as the reference category, 35–49, 50–64, and ≥ 65 years) and educational attainment (less than high school, high school or equivalent, some college/vocational training, and college graduate or higher as the reference category) were categorical variables. We collected disaggregated data on gender identity and race/ethnicity. For analysis purposes, we dichotomized gender (i.e. women as the reference category, and men) and categorized race/ethnicity (i.e. White as the reference category, Black, Asian, Latinx, and Other) rather than using disaggregated data because of small numbers in some groups.

Language preference was reported by the participant and was the language in which the participant completed the survey. If participants reported that they spoke English “not at all”, “poor”, or “not well” [15], English proficiency was defined as limited. Health literacy was asked in reference to materials in participants’ preferred language. Health literacy was reported as limited if participants answered “sometimes”, “often”, or “always” to the question: “How often do you need to have someone help you when you read instructions, pamphlets, or other written material from your doctor or pharmacy?” [11] We decided to use this single-question self-report health literacy item as it has been validated against sentence-completion and vocabulary-based direct health literacy measures in English and Spanish [11, 12]. In addition, it has been used in multiple studies [16–18] instead of burdensome healthy literacy testing.

### Statistical analyses

We calculated descriptive statistics for participants, including means and standard deviations for numeric variables and frequencies and percentages for categorical variables. We assessed differences between language groups using chi-squared tests for categorical variables. We computed weights using iterative proportional fitting (raking), a technique used for nonprobability samples that involves raking over a set of variables (age, gender, and race/ethnicity) iteratively, to reweight the cohort population to match the distribution of the reference population (San Francisco). Among respondents without missing data, we assessed the association between predictor variables and HBV screening using univariable logistic regressions. In addition, we analyzed the association between primary predictor variables (e.g., race/ethnicity, language preference, and a usual place of care) with HBV screening using multivariable logistic regression, adjusting for age, gender, education, English proficiency, health literacy, and insurance status. We determined no statistically significant collinearity or interaction between variables. Given the potential interaction between language preference and nativity, we conducted sensitivity analyses that evaluated models with both language preference and nativity, with each variable alone, and with both variables and their interaction. In these analyses, only language preference was significant, and therefore, our final model only included language preference. We assessed statistical significance at the *p* < 0.05 level for all tests. Stata 16 (College Station, TX) was used for data analysis [19].

## Results

There were 1027 participants who completed the survey, with 50% of surveys administered in English, 25% in Spanish, and 25% in Chinese (Table 1).

**Table 1:** Sociodemographic characteristics of SFHINTS participants by survey language^a^

	Total ***N*** = 1027	English ***N*** = 514	Spanish ***N*** = 256	Chinese ***N*** = 257
Age [mean (SD)]^*^	47.4 (16.7)	44.8 (15.6)	47.0 (15.3)	52.8 (18.9)
Men	536 (48%)	286 (44%)	122 (52%)	128 (50%)
Gender Identity^*^				
Female	537 (52%)	284 (56%)	121 (47%)	132 (52%)
Male	463 (45%)	221 (43%)	118 (46%)	124 (48%)
Female-to-Male (FTM)/Transgender Male/Trans Man	1 (0.1%)	0 (0%)	1 (0.4%)	0 (0%)
Male-to-Female (MTF)/Transgender Female/Trans Woman	13 (1%)	2 (0.4%)	11 (4%)	0 (0%)
Genderqueer, neither exclusively male nor female	8 (0.8%)	3 (0.6%)	5 (2%)	0 (0%)
Additional Gender Category/Other	2 (0.2%)	2 (0.4%)	0 (0%)	0 (0%)
Race/Ethnicity^*^				
White	44 (4%)	43 (8%)	1 (0.4%)	0 (0%)
Black/African American	243 (24%)	242 (47%)	1 (0.4%)	0 (0%)
Latinx	365 (36%)	115 (22%)	250 (98%)	0 (0%)
Asian	317 (31%)	58 (11%)	2 (0.8%)	257 (100%)
Other	58 (6%)	56 (12%)	2 (0.8%)	0 (0%)
Disaggregated Race/Ethnicity^*^				
White	44 (4%)	43 (8%)	1 (2%)	0 (0%)
Black/African American	243 (24%)	242 (47%)	1 (0.4%)	0 (0%)
Latinx	340 (33%)	95 (18%)	245 (96%)	0 (0%)
Asian Indian	3 (0.3%)	2 (0.4%)	1 (0.4%)	0 (0%)
Chinese	295 (29%)	40 (8%)	0 (0%)	255 (99%)
Filipino	7 (0.7%)	7 (1%)	0 (0%)	0 (0%)
Japanese	1 (0.1%)	1 (0.2%)	0 (0%)	0 (0%)
Korean	1 (0.1%)	1 (0.2%)	0 (0%)	0 (0%)
Vietnamese	4 (0.4%)	2 (0.4%)	0 (0%)	2 (1%)
Other Asian	3 (0.3%)	3 (0.6%)	0 (0%)	0 (0%)
Native Hawaiian	0 (0%)	0 (0%)	0 (0%)	0 (0%)
Samoan	15 (1%)	15 (3%)	0 (0%)	0 (0%)
Other Pacific Islander	1 (0.1%)	1 (0.2%)	0 (0%)	0 (0%)
Multi-Race	53 (5%	48 (9%)	5 (2%)	0 (0%)
Other Race	13 (1%)	11 (2%)	2 (1%)	0 (0%)
Highest grade or level of schooling completed^*^				
Less than high school education	236 (23%)	61 (12%)	88 (35%)	87 (34%)
High school graduate or equivalent	305 (30%)	160 (32%)	75 (30%)	70 (28%)
Some college or vocational training	284 (28%)	167 (33%)	59 (23%)	58 (23%)
College graduate or higher	186 (18%)	115 (23%)	31 (12%)	40 (16%)
US born^*^	482 (48%)	424 (84%)	33 (13%)	25 (10%)
Limited English proficiency^*^	344 (34%)	27 (5%)	147 (57%)	170 (66%)
Limited health literacy^*^	426 (42%)	168 (33%)	114 (48%)	144 (57%)
Has health insurance^*^	838 (86%)	404 (83%)	210 (85%)	224 (91%)
At least one usual place of care	849 (88%)	432 (89%)	222 (88%)	195 (87%)
Screened for hepatitis B^*^	521 (51%)	253 (49%)	164 (64%)	104 (41%)

^a^Data were missing for < 10 participants for gender, 13 participants for health literacy; 16 participants for education; 17 participants for their country of birth; 48 participants for presence of insurance; and 66 participants for presence of usual source of care

^*^Significant differences between language groups (*p* < 0.05)

The mean age was 47.4 years (SD 16.7) overall, with Chinese-language respondents being older. Fifty-two percent of all participants were women; 24% identified as Black American, 36% as Latinx, 31% as Asian American, and 4% as non-Hispanic White. Half of participants reported a high school education or lower; English-language respondents were more educated (23% had college graduate degree or higher). Most English-language respondents (84%) were born in the U.S., while a minority of Spanish-language respondents (13%) and Chinese-language respondents (10%) were born in the U.S. A majority of Chinese-language respondents (66%) and Spanish-language respondents (57%) reported limited English proficiency. Most participants had health insurance (86%) and at least one usual place of care (88%). The differences between language groups in terms of age, race/ethnicity, education, nativity, English proficiency, health literacy level, and insurance status were statistically significant.

Only 50% of participants reported HBV screening. HBV screening rates varied by race/ethnicity and language, with 72% for non-Hispanic White respondents, 61% for Latinx respondents, 45% for Black American respondents, 42% for Asian American respondents, and 41% for ‘Other’ race/ethnicity respondents (*p* < .0001). HBV screening rates were highest among Spanish-language respondents (64%) and lowest among Chinese-language respondents (41%) (*p* < .0001).

**Fig. 1:**
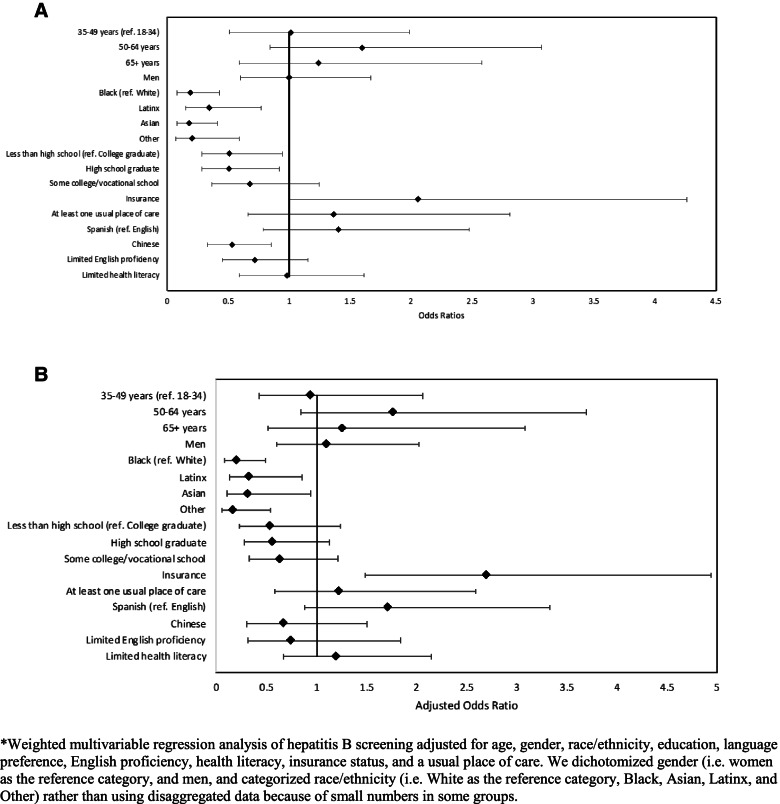
Predictors associated with hepatitis B screening among multilingual, diverse SFHINTS participants. **A**. Unadjusted analysis of predictor of HBV screening. **B**. Multivariable analysis of predictors of HBV screening^*^

In multivariable analysis, race/ethnicity and insurance status were associated with HBV screening (Fig. 1). Black [Odds ratio (OR) = 0.20, 95% confidence interval (CI) 0.08–0.49], Latinx (OR = 0.33, 95% CI 0.13–0.85), Asian (OR = 0.31, 95% CI 0.10–0.94), and ‘Other’ race/ethnicity (OR = 0.17, 95% CI 0.05–0.53) respondents had lower odds of HBV screening compared to non-Hispanic White respondents. Participants who had insurance had increased odds of HBV screening (OR = 2.70, 95% CI 1.48–4.93). Education and Chinese language preference were associated with HBV screening in unadjusted but not in multivariable analysis. Having a usual place of care was not associated with HBV screening.

## Discussion

In a diverse, multilingual sample, HBV screening rates were sub-optimal but comparable to prior studies [5]. Despite efforts over the past few decades to increase screening, including guidelines for high-risk groups [20], HBV continues to be under-recognized by those infected and under-diagnosed by their health care professionals. Importantly, even after adjusting for language preference, health literacy level, and having a usual place of care, HBV screening disparities persist for Black Americans, Asian Americans, Latinx, ‘Other’ race/ethnic groups, and the uninsured. The suboptimal rates of HBV screening in our study suggest that current strategies to increase screening have not been implemented or have not been successful, particularly in terms of health equity. Interventions engaging lay healthcare workers in community-based HBV screening [21–23] and leveraging information technology to enhance clinical decision-making tools for providers [24] have been effective in increasing rates of HBV screening. Expanding insurance coverage and considering a one-time universal screening for chronic HBV [25] may improve rates of HBV screening, particularly in high-risk communities.

Even though an effective vaccination and treatment exists for HBV [20], these interventions are only successful if individuals with HBV are accurately identified and linked to appropriate care and treatment. Understanding and intervening upon barriers to HBV screening, particularly for vulnerable populations who are at higher-risk of chronic HBV infections, can also help reduce disparities in HBV-related complications, including cirrhosis and liver cancer [5]. Improving HBV screening rates, reducing the number of new HBV infections, and ensuring appropriate monitoring and care for patients with HBV may reduce the morbidity and mortality related to HBV, which remains a significant public health problem [8, 26].

Our study has several limitations. First, the survey was administered in one geographic location and may not be generalizable to other areas. However, in order to inform local efforts to address cancer disparities, we prioritized this diverse, urban, low-income population. In addition, as a cross-sectional study, we were limited in our ability to assess trends in HBV screening over time. Furthermore, HBV screening was based on self-report. HBV screening rates could have been overestimated if participants conflated HBV screening with HBV vaccination or general health screening. Participants may also have underreported HBV screening, given potential stigma related to having HBV, as highlighted by about 20% of patients not knowing or refusing to answer whether or not they have had HBV. However, the overall screening rates reported herein, and our finding of lower screening rates among racial/ethnic minorities, are consistent with prior literature [1, 3]. Finally, we did not have access to behavioral variables such as injection drug use, high-risk sexual behavior, immunosuppressive therapy use, or HIV status, which may have impacted why patients were screened for HBV. Future studies should explore the impact of these key variables in underserved, high-risk populations.

Future studies should explore reasons why current strategies have not been implemented or are not successful, particularly in addressing racial/ethnic and insurance disparities. Potential strategies to address disparities in HBV screening for diverse communities include addressing stigma, implementing automated tools within the electronic medical record to reduce healthcare provider burden, re-envisioning patient and community education and engagement, and expanding insurance coverage.

## Conclusion

Prior studies have shown disparities in chronic HBV infection and associated complications. While HBV screening can prevent morbidity and mortality related to chronic liver disease, many people are still unaware of being infected. This study’s focus on low-income, communities of color in San Francisco, who are disproportionately burdened by chronic HBV infection, allowed us to explore factors associated with HBV screening that may lead to targeted interventions to reduce disparities in HBV-related outcomes in these vulnerable populations. We found that while HBV screening rates are sub-optimal for all populations, HBV screening disparities persist for Black Americans, Asian Americans, Latinx, ‘other’ race/ethnic groups, and the uninsured. Future studies need to explore the potential individual, health system, and structural level factors that serve as barriers to HBV screening, particularly in high-risk communities.

## Supplementary Information

**Additional file 1.**

**Additional file 2.**

## Data Availability

The datasets used for the study are available from the corresponding author upon reasonable request.

## Abbreviation

HBV: Hepatitis B Virus
