# Supplementary material for: Exploring factors associated with hepatitis B screening in a multilingual and diverse population
Source: BMC Health Serv Res. 2022 Apr 11;22:479. doi: 10.1186/s12913-022-07813-w (PMC8996655; doi:10.1186/s12913-022-07813-w)
Supplement: Supplementary file 2 — Additional file 2. [file 12913_2022_7813_MOESM2_ESM.pdf]

Appendix B: Health Behavior Framework[a] describes individual variables; provider and health care system factors; and individual, system, and societal barriers and supports for hepatitis B screening

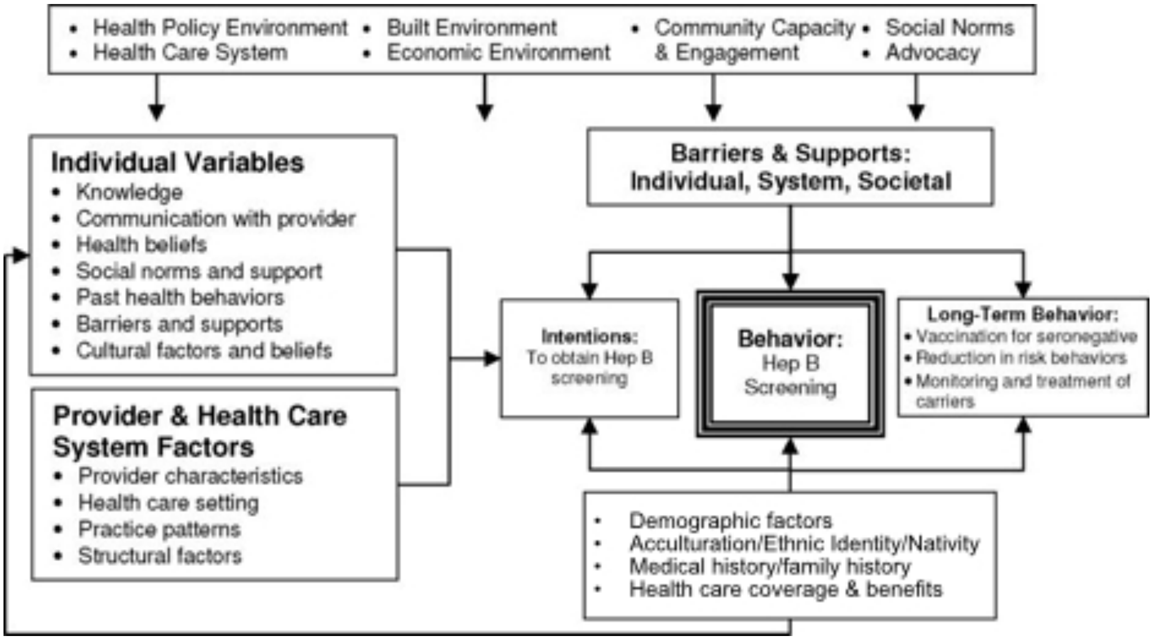

[a]Adapted from: Bastani R, Glenn BA, Taylor VM, Chen MS Jr, Nguyen TT, Stewart SL, Maxwell AE. Integrating theory into community interventions to reduce liver cancer disparities: The Health Behavior Framework. Prev Med. 2010 Jan-Feb;50(1-2):63-7. doi: 10.1016/j.ypmed.2009.08.010. Epub 2009 Aug 27.
